# Epigenetic origin of evolutionary novel centromeres

**DOI:** 10.1038/srep41980

**Published:** 2017-02-03

**Authors:** Doron Tolomeo, Oronzo Capozzi, Roscoe R. Stanyon, Nicoletta Archidiacono, Pietro D’Addabbo, Claudia R. Catacchio, Stefania Purgato, Giovanni Perini, Werner Schempp, John Huddleston, Maika Malig, Evan E. Eichler, Mariano Rocchi

**Affiliations:** 1 Department of Biology, University of Bari, Bari, Italy; 2 Department of Biology, University of Florence, 50122 Florence, Italy; 3 Department of Pharmacy and Biotechnology, University of Bologna, Bologna, Italy; 4 Institute of Human Genetics, University of Freiburg, 79106 Freiburg, Germany; 5 Department of Genome Sciences, University of Washington School of Medicine, Seattle, WA 98195, USA; 6 Howard Hughes Medical Institute, University of Washington, Seattle, WA 98195, USA

## Abstract

Most evolutionary new centromeres (ENC) are composed of large arrays of satellite DNA and surrounded by segmental duplications. However, the hypothesis is that ENCs are seeded in an anonymous sequence and only over time have acquired the complexity of “normal” centromeres. Up to now evidence to test this hypothesis was lacking. We recently discovered that the well-known polymorphism of orangutan chromosome 12 was due to the presence of an ENC. We sequenced the genome of an orangutan homozygous for the ENC, and we focused our analysis on the comparison of the ENC domain with respect to its wild type counterpart. No significant variations were found. This finding is the first clear evidence that ENC seedings are epigenetic in nature. The compaction of the ENC domain was found significantly higher than the corresponding WT region and, interestingly, the expression of the only gene embedded in the region was significantly repressed.

The centromere is the chromosomal structure that ensures proper segregation of chromosomes during mitosis and meiosis. In most eukaryotes the centromere is embedded in a complex structure composed of arrays of satellite DNA often flanked by clusters of segmental duplications. The discovery and characterization of *de novo* centromeres in humans and other species has challenged the necessity of this sequence complexity for proper centromere function (for a review see Marshall *et al*.^1^). These *de novo,* ectopic neocentromeres, which have now been described in dozens of medical genetic reports are devoid of satellite DNA, yet are fully functional. They form in apparently random, anonymous sequence, usually to stabilize acentric chromosomal fragments. The negative phenotypic consequences and reduced fitness of these supernumerary chromosomes often bring these neocentromere cases to clinical observation. In a few cases the individual and karyotype are normal except that the centromere has shifted to a new location along the chromosome. These serendipitously discovered new centromeres are also devoid of satellite DNA.

In 1999, while studying the evolution of chromosome 9 in Old World monkeys (OWM), we discovered the first clear cases of a repositioned centromere fixed in primate species^2^. The phenomenon involved the movement of the centromere along the chromosome without a change in the marker order. We coined the term “evolutionary new centromere” or ENC to distinguish these from evolutionary conserved centromeres. Subsequent research revealed, to our surprise, that ENCs occurred relatively frequently not only in primates, but also in many other vertebrate species (for review see Rocchi *et al*.). ENCs have recently been reported in plants^3^,^4^. In macaques, 9 out of 20 autosomal centromeres were found to be ENCs that evolved in the ancestor of OWM^5^. However, all these ENCs accommodated large blocks of alphoid sequences that make them, at least on this level, undistinguishable from other centromeres. Because these ENCs are shared by all OWM species^5^, they must be at least 16 million years old before the split of the Cercopithecinae/Colobinae^6^.

The data from clinical studies of neocentromeres support the hypothesis that ENCs were also initially seeded in anonymous sequences devoid of satellite DNA. A second, but correlated hypothesis, is that the satellite DNA of mature centromeres was acquired only secondarily, overtime. These hypotheses are supported by the discovery that the numerous ENCs in equids were “nude”, devoid of satellite DNA^7^,^8^,^9^. In plants, “nude” centromeres, hypothesized to be ENCs, have been reported in *Solanum* species^3^,^10^,^11^ and in maize^4^.

*Equus* species shared a common ancestor about 2–3 million years ago^12^, so these ENCs are relatively young. They, however, are fixed in the population, making it difficult to track potential changes that may have occurred since their seeding. Until now, a clear test of this hypothesis was not possible, because data on the seeding of ENCs and the maturation process were lacking.

Early comparative cytogenetic studies described a polymorphic orangutan chromosome 12 (chromosome 9 of classical nomenclature)^13^,^14^,^15^ showing high allele frequency (over 20%) in both Borneo (*Pongo pygmaeus*, PPY) and Sumatra (*Pongo abelii*, PAB) orangutan populations^16^. Recently we showed without doubt that the marker order of the variant chromosome was identical to the normal homolog, and that the polymorphism was due to a centromere repositioning event, that is it was a further example of a primate ENC (hereafter referred to as ENC12)^17^. This ENC is unique because both the original centromere and the new centromere coexist in the same population as a polymorphism. Further, ChIP-on-chip analysis, performed on a heterozygous individual (PPY-10), showed that the neocentromeric domain mapped at ~chr12:85,205,000-85,430,000 (ponAbe2 assembly, UCSC genome browser)^17^. We argued that this ENC was relatively young due to its lack of complex satellite DNA. Its presence in both Bornean and Sumatran orangutans indicates that it predates their divergence estimated to have occurred 400,000 years ago^17^.

These considerations prompted us to investigate the ENC12 sequence and structure in more detail in an attempt to identify the initial steps of the maturation process of an ENC. The results show that no changes at the sequence level had occurred, but that the ENC domain was significantly more compacted compared to its wild type (WT) counterpart on the normal chromosome, and that the expression of *SLC6A15*, the only gene embedded in the region, was significantly repressed.

## Results

**Figure 1:**
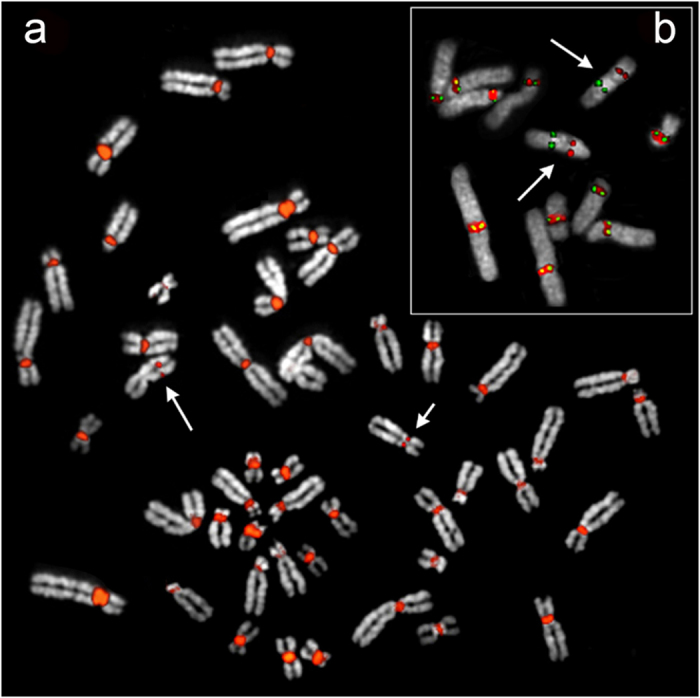
Immuno-FISH characterization of the orangutan ENC12. (**a**) The metaphase is from PPY-10, heterozygous for the ENC12, hybridized with a pool of alphoid centromeric sequences obtained as described in Methods (red signal). The short arrow indicates the normal PPY12, while the long one points to the ENC12 chromosome. (**b**) The partial metaphase is from the PPY-15 homozygous. The two ENC12 chromosomes (arrowed) show the FISH signal of the centromeric alphoid sequences (red) at the old deactivated centromere and the immuno signal (green) at the functional ENC12 centromere. The immuno signal was obtained using antibodies against the centromeric protein CENP-C.

To facilitate analysis of the ENC of orangutan chromosome 12, we searched for an orangutan that was homozygous for the neocentromere. The screening was conducted by karyotyping banded chromosomes of a series of orangutans. After screening 16 orangutan lymphoblastoid cell lines (8 Sumatran and 8 Bornean orangutans) we found six heterozygous orangutans (4 PAB and 2 PPY) and one homozygous individual (PPY-15). These results were confirmed by fluorescence *in situ* hybridization (FISH), as illustrated in Fig. 1. All lymphoblastoid cell lines were derived from captive animals. Their assignment to Sumatran or to the Bornean species was confirmed cytogenetically by the presence of the Borneo-specific pericentric inversion of chromosome 3 (classical nomenclature: orangutan 2)^18^ (see http://www.biologia.uniba.it/orang/PPY/PPY_03.html).

### Precise mapping of the ENC

**Figure 2:**
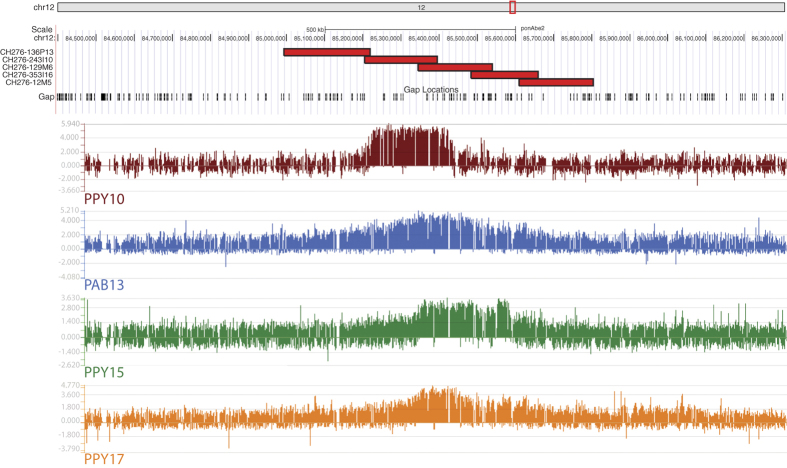
ENC12 CHIP-on-chip results and mapping of the sequenced BACs. The Figure graphically reports the ChIP-on-chip results. DNA obtained by chromatin immunoprecipitation, using an anti-CENP-A antibody, was hybridized to a tiling array covering the neocentromeric region. Results are presented as the log2 ratio of the hybridization signals obtained with immunoprecipitated DNA versus input DNA. The Figure also reports the position of the five CH276 BAC clones that were PacBio sequenced (see the following paragraphs), with respect to the ponAbe2 sequence, and to the ChIP-on-chip results.

As mentioned, we already obtained a precise mapping of the functional ENC12 of a heterozygous orangutan (PPY-10) by ChIP-on-chip analysis using antibodies against the centromeric protein CENP-A^17^. We performed ChIP-on-chip experiments in three additional orangutan individuals, one homozygous (PPY-15) and two heterozygous (PAB-13 and PPY-17) (Arrays data have been deposited to the NCBI’s Gene Expression Omnibus and are accessible through GEO Series accession number GSE81003, https://www.ncbi.nlm.nih.gov/geo/query/acc.cgi?acc=GSE81003). The results (Fig. 2) revealed that the total ENC functional domain spanned a region of about 605 kb (~chr12:85,070,000-85,675,000).

### Long Range PCR analysis

In order to assess potential gross differences between the ENC12 sequence domain and the corresponding WT region, we designed, using the ponAbe2 orangutan assembly, a panel of 55 primer pairs for long range PCR (LR-PCR) experiments (average LR-PCR length: 6.2 kb), spanning 599,371 of the 775,997 bp defined by the first and last primer (chr12:84,995,606-85,771,603; primers in Supplementary Table S1). Each experiment was performed on DNA from three orangutan lymphoblastoid cell lines: WT homozygous PAB-9, the heterozygous individual PPY-10, and the PPY-15, homozygous for the ENC. Amplification products were obtained for 45 of these primer pairs. In all cases the products yielded, on agarose gel, a single band identical in all the three individuals irrespective of ENC status. The 10 amplification failures were due, very likely, to the low quality of the ponAbe2 sequence, full of large gaps. Primers could be misoriented or their distance underestimated.

### ENC12 domain sequence in the WT and in the homozygous individual

To overcome the limitations of the ponAbe2 assembly, we identified five overlapping orangutan BACs from the BAC library CH276 (CH276-136P13, −243I10, −129M6, −353I16, and −12M5) spanning ~810 kb (ponAbe2 chr12:84,993,491-85,804,188) and centered on the ENC12 domain (see Fig. 2). The CH276 library was derived from the same female individual (Susie) used for the orangutan sequencing project, homozygous for the normal centromere^17^. We sequenced the five BACs using the single-molecule real-time (SMRT) sequencing technology (Pacific Biosciences, Menlo Park, CA) and generated high quality sequence for each of them using previously described methods^19^. The long reads for the BAC assemblies were filtered using default HGAP parameters as described^19^. We used these finished sequences to generate a new reference sequence for the region, hereafter referred to as PacBio805 (805,775 bp in length; deposited in GenBank under the Accession Number KX224531). *In silico* testing of the failing 10 primer pairs (see above) against PacBio805 sequence confirmed that the ponAbe2 reference sequences were or incorrectly oriented, or too distantly positioned, or that the primer pair-binding site had too many mismatches or no match when compared to the PacBio805 sequence. We designed 31 additional primer pairs using the PacBio805 sequence and performed additional LR-PCR experiments on PAB-9, PPY-10, and PPY-15 (primers in Supplementary Table S1). All the experiments were successful and the lengths of the amplified products, always identical in the three samples, were all concordant with the predicted distance of the primers in the PacBio805 sequence.

**Figure 3:**
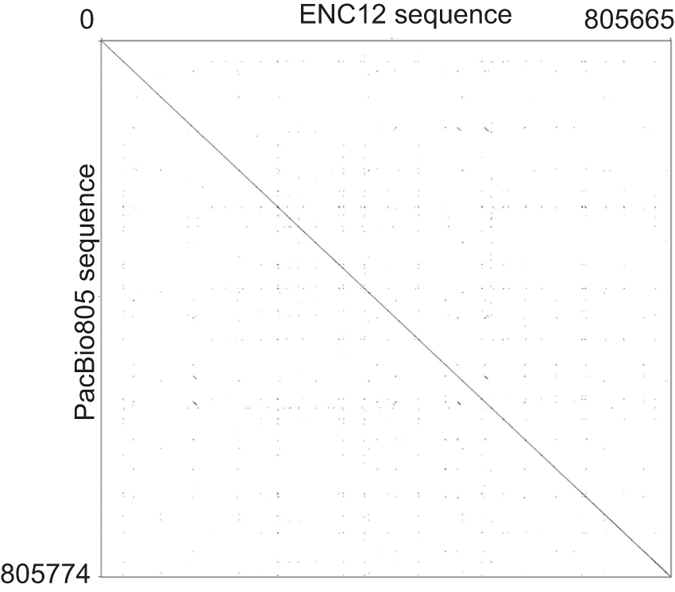
Sequence comparison of the ENC12 domain versus WT. Dot plot matrix comparing PacBio805 sequence (Y axis) to the corresponding region in NGS12 sequence (ENC12, X axis), using Gepard-1.40^56^. Sequence lengths are given at the axis ends.

In order to characterize the ENC12 functional centromeric domain corresponding to the homozygous individual, we performed whole genome sequencing of the NEO12 homozygous PPY-15 cell line, using NGS Illumina HiSeq 2500 technology. We obtained a total coverage of 46X of the orangutan genome (50.8X coverage of the region corresponding to the PacBio805). We then generated an assembly of chromosome 12 (hereafter referred to as NGS12) (NGS reads of the orangutan PPY-15 are available at http://www.ebi.ac.uk/ena/data/view/PRJEB13951. The NGS12 assembly is available at http://www.ebi.ac.uk/ena/data/view/LT571452-LT571452; see Materials and Methods for details of sequence production and quality control). To validate the quality and structure of the ENC domain within the NGS12 assembly, we sequenced, using Sanger sequencing technology, 106 paired-ends of the LR-PCR products from PPY-15 (see Supplementary Table S1 for primers; all the 106 paired-end sequences have been submitted to GenBank under the provisional Accession Number KX243426 - KX243531). The end sequences (86 kb in total) were distributed evenly along the neocentromeric domain (chr12: 84,995,606- 85,771,603) (Supplementary Fig. S1) and represented over 10% of the segment covered by the entire PacBio805 sequence. Sequence alignment between the LR-PCR paired ends and ENC12 showed an average of 99.6% identity with a mode of 100% (Supplementary Table S1), confirming, *in toto*, that our sequence assembly of the ENC12 domain was high quality. We then compared the sequence of the PacBio805 to the corresponding region of the NGS12 sequence using blast2seq^20^. The identity between the two was high (99.6% or 803,027/806,142 bp) (Fig. 3). Details of the discrepancies between the two sequences are reported in Supplementary Table S2.

### Mobile elements of the ENC12 region

Mobile elements frequency in the PacBio805 sequence, which represents the seeding region of the ENC12 centromere, was compared to the entire NGS12 sequence using RepeatMasker software^21^. Both sequences were screened for interspersed repeats and low complexity DNAs. The analysis revealed no significant differences. In detail, in PacBio805 and ENC12 total interspersed repeats (LINEs, SINEs, LTR elements and DNA elements) spanned 47.19% and 47.36%, respectively; simple repeats 1.30% and 1.36%, respectively. Low complexity repeats value was 0.26% in both sequences.

### Genes mapping within the ENC12 domain

*SLC6A15* (chr12:85,450,585-85,510,678) is the only gene mapping within the ENC12 domain (*Pongo abelii* solute carrier family 6, neutral amino acid transporter, member 15). The most close orangutan RefSeq genes on telomeric and centromeric sides of the ENC12 domain are *METTL25* (chr12:82,804,725-82,919,534), *Pongo abelii* methyltransferase like 25, and *C12H12orf29* (chr12:88,851,588-88,866,204) *Pongo abelii* chromosome 12 open reading frame, human *C12orf29*. They are ~2,280 kb and ~3,272 kb apart from the centromeric and telomeric side of the ENC12, respectively.

Ensemble lists 5 different paralogs of the *SLC6A15* gene: *SLCA17* on chromosome 1, *SLC6A16* on chromosome 19, *SLC6A20* on chromosome 20, *SLCA18* on chromosome 5, and *SLCA19*, also on chromosome 5. We measured the Residual Variation Intolerance Score (RVIS)^22^ of the *SLC6A15* gene and its paralogues in order to evaluate its disposability. Only *SLC6A15*, along with *SLC6A18*, have positive RVIS values (0.07 and 1.01, respectively).

In order to evaluate the *SLC6A15* expression we performed RT-qPCR (Quantitative reverse transcription PCR) experiments on RNA from lymphoblastoid cell lines of PAB-9 (homozygous normal) and PPY-15 (ENC12 homozygous). Although *SLC6A15* is poorly expressed in lymphoblastoid cells, the analysis revealed a definite lower expression level of the gene in the PPY-15 with respect to the PAB-9 normal individual. The difference was significant for three out of four tested primer pairs (*p* < 0.05) (Supplementary Fig. S2 and Table S3).

### Compaction of the ENC region with respect to the WT counterpart

Centromeric domains are usually more compacted with respect to the euchromatic portion of the genome^23^,^24^,^25^. Compaction, however, appears to be a general feature of heterochromatin^26^. We wanted to test the hypothesis that the compaction is an intrinsic property of active centromeres even when they are devoid of satellite DNA. The analysis was also performed because the compaction status of the region could provide an explanation for the low expression of the *SLC6A15* gene.

**Figure 4:**
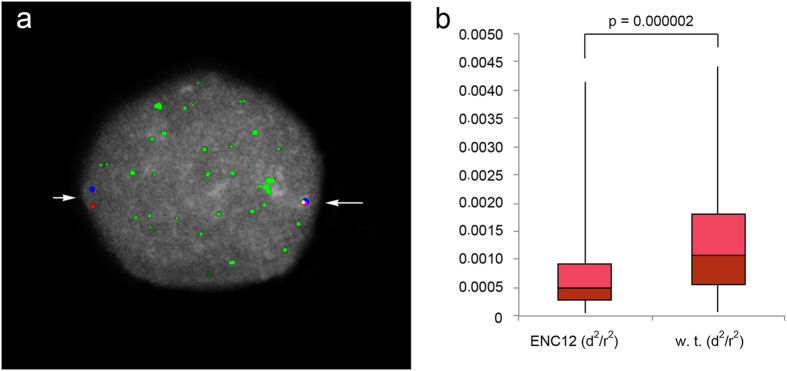
ENC chromatin compaction. (**a**) Example of an immuno-FISH experiment on an interphase nucleus of the heterozygous PPY-10 individual. The compaction was measured using the BACs CH276-136P13 (red signal) and CH276-12M5 (blue signal), flanking the ENC12. Their precise position on the ponAbe2 assembly is reported in the Fig. 2. The green immuno signal of CENP-C was used to discriminate the ENC12 domain (long arrow) from the WT counterpart (short arrow). (**b**) The boxplot shows the distributions of interprobe normalized distances for the ENC12 and its WT counterpart. *P*-value was calculated by Mann-Whitney U-tests.

Mean-squared interprobe distance (d^2^) is known to be linearly related to genomic distance at probe separation <2 Mb^27^,^28^. We performed immuno-fluorescence *in situ* hybridization (immuno-FISH) experiments using the orangutan BACs CH276-136P13 and CH276-12M5 (chr12:84,993,491-85,219,222 and chr12:85,610,154–85,804,188, respectively), flanking the ENC12 and positioned ~391 kb apart. These two BACs are the most external of the 5 BACs that were sequenced using SMRT sequence technology (see Fig. 2). We performed these experiments on serum-starved lymphoblastoid cells of the heterozygous individual PPY-10. The ENC12 centromeric domain and the WT homologous region were easily discriminated by the immuno-signal due to the antibodies against the centromeric protein CENP-C (example in Fig. 4). The heterozygosity of PPY-10 ensured that the compaction measures of the ENC12 and its normal counterpart were obtained under identical technical conditions. Additionally, the bias derived from variations in nuclear size was silenced by normalizing d^2^ by the nuclear radius (r^2^) visualized by DAPI staining^29^,^30^. The statistical analysis performed on 100 interphase nuclei showed that the ENC12 domain was significantly more compacted than the corresponding normal region (p value ≪ 0.01 in Mann-Whitney U analysis).

## Discussion

ENCs were frequently found in evolutionary studies of mammalian chromosomes. The majority are ancient and have acquired the satellite DNA complexity of normal mature centromeres^31^. A similar evolutionary trend was proposed for plants^3^,^4^,^10^,^11^. The hypothesis that ENC are epigenetically seeded stems from several indirect lines of evidence: (i) dozen of human “clinical” neocentromeres, some of them fully sequenced, do not have satellite DNA and (ii), do not have any sequence similarity or significant shared features^1^; (iii) similar observations were also reported for human cases of neocentromeres that repositioned along the chromosome, without phenotypic effects and with the potential to spread in the population, thus providing a model mechanism for ENC seeding and development^32^,^33^,^34^,^35^,^36^,^37^,^38^,^39^; (iv) relatively young, fixed, and satellite-free ENC have been reported in equids^7^,^8^,^9^. We can also note that even independent neocentromeres that appeared, cytogenetically, to arise at the same hot-spot, were shown at the sequence level to have different locations^40^,^41^.

One important result of our study is that the sequence underlining the ENC appears unchanged as compared to the WT counterpart even after a minimum of 400,000 years. We conclude that the domain did not undergo any sequence changes due to ENC seeding. This unique finding strongly supports the hypothesis that ENC formation is epigenetic in nature. The ENC12 is present at high frequencies in both Sumatra and Borneo orangutans, strongly indicating that its seeding happened in their common ancestor before their divergence, which occurred at least 400,000 ya, even if the exact age of the ENC cannot be precisely determined.

The available data in other species indicated that, after seeding, ENCs then acquire over time the normal complexity of mature centromeres (arrays of centromeric satellite DNA surrounded by segmental duplications)^3^,^4^,^5^,^10^,^11^,^31^. This hypothesis implies a profound restructuring of the region. Our finding indicates that this process, very likely, does not start immediately after seeding, and that it does not necessarily consists of a gradual accrual of satellite DNA and/or small accumulations of sequence variations. It can be hypothesized that the changes may be discontinuous and discrete, perhaps occasionally initiated at the centromere clustering that occurs at leptotene in diploid organisms^42^. This initial event of centromeric satellite acquisition could then trigger a cascade of subsequent changes leading to centromere maturation.

Previously we showed in primates that ENCs preferentially occur in gene deserts^43^. In plants the situation appears more complex, but substantially supports this conclusion (for review see Wang *et al*.^4^). The hypothesis is that an ENC seeded in a gene desert has a higher chance of avoiding negative selective effects, and eventually becomes fixed in the population. Further, we know that ENCs almost always then undergo a deep restructuring, for example as demonstrated by macaque chromosome 6^5^. Restructuring is regarded as potentially detrimental to the normal function of genes embedded in the region, which could then inhibit ENC spreading in the population. The present results provide essential information to test this hypothesis. Expression of the *SLC6A15* gene, the only gene embedded in the ENC region, appears repressed, very likely as the consequence of the regional compaction. The bias against ENC fixation could therefore initiate long before the appearance of satellite DNA and the restructuring of the region. In the present case this scenario was disclosed because the *SLC6A15* gene appears to be disposable, and thus does not act as a bias against the ENC spreading in the population. Nevertheless, Saffery *et al*.^44^ did not detect gene expression negative interference in a human neocentromere. However, the investigation was performed in a somatic cell hybrid in which the neocentromere was isolated. Negative interference was reported in chickens^45^, and data from plants substantially support this view^4^,^10^.

The significant compaction of the neocentromeric domain with respect to the WT counterpart is an additional important, general result of our study. Our results show that compaction is an intrinsic property of centromeres, independently from the presence of heterochromatin. If compactness affects gene expression, it could inhibit ENC spreading. This effect would be dependent on the importance of the gene(s). The incomplete repression of the *SLC6A15* gene however raised the possibility that it might not be completely disposable. An alternative hypothesis might be that the activity of the gene has helped maintain this area in its original satellite free condition since its seeding.

Data presented here provide strong evidence that ENC seeding is epigenetic in nature, that centromere formation exerts a substantial compaction irrespective of the presence of satellite DNA, and that the compaction affects gene expression. However, hypotheses on the subsequent maturation process need further research. The tempo and mode of satellite and repeat DNA accumulation could be clarified by studying a phylogenetic array of ENCs at different ages and maturation stages. The role played by the presence or absence of genes in the centromeric domain on the accumulation of repeat DNA sequences could be clarified by the discovery and comparison of new ENC cases. On the basis of the relatively high number of ENCs reported in literature, such task should be within reach. Sequencing entire genomes has become a routine task, and papers on sequenced genomes frequently report data on population variability. Sequencing technologies, however, are not able to detect, or even hypothesize the existence of an ENC devoid of satellite DNA. If such population studies are implemented, molecular cytogenetic studies, therefore, will continue to be an essential complement to sequencing data.

## Methods

### ChIP and ChIP-on-chip analysis

To identify the sequences bound by CENP-A, native chromatin immunoprecipitation analysis was performed, as previously described^17^. Briefly, lymphoblastoid cell lines from orangutan were processed and native chromatin was prepared by nuclease digestion of cell nuclei; immunoprecipitation was then performed using a polyclonal antibody against the centromeric protein CENP-A^46^. We have previously demonstrated that this antibody is able to recognize horse^7^ and orangutan^17^ centromeres. Both input and immunoprecipitated DNA fragments were purified and amplified using the whole genome amplification kit (Sigma-Aldrich, St. Louis, USA). ChIPed DNA was analyzed by real-time PCR before and after whole genome amplification using the following primers (ponAbe2 release):

PPY SAT F: AGTGTTTCAAAACTGCTCTA (satellite DNA).

PPY SAT R: CTTTTTGTAGAATCTGCAAGT (satellite DNA).

PPY 1p1 F: GGCAGCTGGTAACAAAACAGA (chr12:85,251,142-85,251,162).

PPY 1p1 R: TTTGTTCATTCCCGTTTCAG (chr12:85,251,207-85,251,226).

PPY 1p2 F: GACTTTCCTGGGGAAAACCT (chr12:85,315,599-85,315,618).

PPY 1p2 R: AAATCGTCATGCTCCCTCAG (chr12:85,315,688-85,315,707).

PPY no down F: AAACCCCCTGCAAAAACTTC (chr12:86,110,873-86,110,892).

PPY no down R: AGGTCCTTTGCTGCATCAGT (chr12:86,110,943-86,110,962).

The input and the immunoprecipitated DNAs were cohybridized to a NimbleGen custom tiling array containing a 5 Mb centered in the ponAbe2 sequence (chr12: 82,500,000-87,500,000) with an average resolution of 142 bp. DNA binding peaks were identified by using the statistical model and methodology described at (http://chipanalysis.genomecenter.ucdavis.edu/cgi-bin/tamalpais.cgi)^47^ using stringent parameters for peak identification (98th percentile threshold and p < 0.0001).

### Long-range PCR

The long-range PCR analyses were carried out using a commercial kit (TaKaRa LA Taq™; Cambrex Bio Science, Milan, Italy) according to the manufacturer’s instructions. All the experiments were performed on genomic DNA extracted from the PAB-9, PPY-10 and PPY-15 cell lines, using the gSYNC Mini kit (Geneaid Biotech Ltd, New Taipei City, Taiwan). Primers (Supplementary Table S1) were designed with Primer3Plus software^48^ on the ponAbe2 genome reference and the PacBio805 sequence. In each reaction, 15 pmol of each primer and 100 ng of template DNA were included. The amplifications were carried out in a T100 thermal cycler (BIO-RAD, California, USA) at the following temperature profiles: 94 °C for 4 min; 8 cycles of 94 °C for 30 s, 60 °C for 30 s, and 68 °C for 8 min; 28 cycles of 94 °C for 30 s, 60 °C for 30 s, and 68 °C for 8 min + 10 s/cycle; 68 °C for 10 min; and 4 °C for the remainder of the reaction. The obtained products were separated on 1% agarose gel, purified by QIAquick PCR purification kit (Qiagen, Hilden, Germany), and sequenced at BMR genomics (Padova, Italy) by Sanger method. Sequence alignment between the LR-PCR products and ENC12 were performed by blast2seq software^49^.

### DNA library preparation and massively parallel sequencing

‘TruSeq DNA PCR free library preparation kit’ (Illumina, San Diego, CA) has been used for library preparation following the manufacturer’s instructions, starting with 3 μg of good quality DNA as input. DNA was sheared by Bioruptor (Diagenode) to obtain fragments of ~250 bp. Libraries were quantified and quality tested using the Qubit 2.0 Fluorometer (Invitrogen, Carlsbad, CA) and Agilent 2100 Bioanalyzer (Agilent Technologies, Santa Clara, CA). Sequencing was performed on Illumina’s HiSeq 2500 ‘High Output’ system generating 132 bp paired-end data. The CASAVA 1.8.2 version of the Illumina pipeline was used to process raw data for both format conversion and de-multiplexing.

### Sequence alignment, variant calling, and annotation

The 1.36 G produced paired-end reads were first trimmed in order to remove lower base quality data with ERNE^50^ and adapter sequences with Cutadapt^51^. Alignment on the *Pongo abelii* reference genome (ENSEMBLE, PPYG2, release 78) was performed with BWA^52^, resulting in 97.23% of aligned reads. Alignment was sorted with SAMTools^52^ and processed with picard-tools (http://broadinstitute.github.io/picard/). Only reads with mapping quality higher than 10 were selected with SAMTools^52^. PCR and optical duplicates were removed from all alignments with picard. Overall mean coverage was 46X; 13.5% of genome has not been covered. Estimated insert size of the library was 341 ± 50 bp.

Variant (SNP and indel) calling and genotyping was performed with GATK^53^. GATK local realignment tool was used to locally realign reads and minimize the number of mismatching bases across all the reads. GATK tools UnifiedGenotyper and Variant Filtration were used for variant calling and for hard-filtering of variant calls based on following criteria: filter out variants if there are at least four alignments with a mapping quality of zero (MQ0) and if the proportion of alignments mapping ambiguously corresponds to 1/10^th^ of all alignments [MQ0 > = 4 && ((MQ0/(1.0 * DP)) > 0.1)], DP: total (unfiltered) depth over all samples; filter out variants which are covered by less than 10 reads [DP < 10]; filter out variants having a low quality score [Q < 50]; filter out variants with low variant confidence over unfiltered depth of non-reference samples (QD) [QD < 1.5]; filter out variants based on strand bias (SB) [SB > −10.0]. All variants were annotated by Annovar^54^.

### Comparison to the BAC sequence

Reads that mapped to the *Pongo abelii* region chr12:84,993,491-85,804,188 were extrapolated from the alignment BAM file and were aligned to the BAC sequence using BWA^52^. 312,074 reads mapped to BAC sequence with the resulting mean coverage of 50.8X. All of the subsequent bioinformatics analysis procedure as well as variant calling and filtering parameters were the same as those described in *Sequence alignment, variant calling, and annotation* paragraph.

### Immuno-FISH

Immunofluorescence using CENP-C antibody was performed on standard preparations according to Earnshaw and Tomkiel^55^ with minor modifications. Distances between BACs CH276-136P13 and CH276-12M5 were measured on interphase nuclei of lymphoblasts serum-starved for 36 hours before harvesting. Cell preparations were stored in a fixative solution (methanol and acetic acid, 3:1) at −20 °C and few drops were used to prepare each slide. As soon as the surface was dry, each slide was rehydrated by immersion in 1X PBS-Azide (10 mM NaPO_4_ at pH 7.4, 0.15 M NaCl, 1 mM EGTA, 0.01% NaN_3_) for 15 min at room temperature. The chromosomes were then swollen by washing the slides three times (2 min each) with 1X TEEN (1 mM treithanolamine-HCl at pH 8.5, 0.2 mM NaEDTA, 25 mM NaCl), 0.5% Triton X-100, 0.1% BSA. The primary polyclonal antibody against the centromeric protein CENP-C was diluted 1:40 in the same solution and then added (100 μL) on the surface of the slide. Each slide was incubated for 2 h at 37 °C. Unlabeled primary antibody was removed by washing the slides at room temperature three times (2, 5 and 3 min) with 1X KB buffer (10 mM Tris-HCl at pH 7.7, 0.15 M NaCl, 0.1% BSA). Secondary antibody conjugated with FITC was diluted 1:40 in the same solution and 100 μL were then added to the slide, and incubated 45 min at 37 °C in a dark chamber. Following incubation with the secondary antibody, the slide was washed once with 1X KB for 2 min, prefixed with 4% paraformaldeide in 1X KB for 45 min, washed with distilled H_2_O by immersion for 10 min at RT, and fixed with methanol and acetic acid (3:1) for 15 min. After that, the standard procedure was followed for FISH.

FISH experiments were performed using BAC clones directly labeled by nick-translation with Cy3-dUTP and Cy5-dUTP. Briefly: hybridization was performed at 37 °C in 2X sodium chloride sodium citrate (SSC), 50% (v/v) formamide, 10% (w/v) dextran sulfate, 3 μg C0t-1 DNA, and 3 mg sonicated salmon sperm DNA, in a volume of 10 μl. Post hybridization washing was at high stringency conditions (60 °C in 0.1x SSC, three times). Nuclei and chromosome metaphases were DAPI-stained. Digital images were obtained using a Leica epifluorescence microscope equipped with a cooled CCD camera. Fluorescence signals detected with Cy3, Cy5 and FITC filters and chromosomes and nuclei images detected with DAPI filter were recorded separately as grayscale images. Pseudocoloring and merging of images were performed using Adobe Photoshop software.

The alphoid array present on PPY12 is very tiny and difficult to detect by FISH (see Supplemental Fig. S8–5 reported in^17^. The FISH experiment reported in Fig. 1 was obtained by labeling PCR products using the primers Alpha3for (TCAACTCTGTGAGATGAATGCAAAC) and Alpha4rev (AAACATCTTTGTGATGTGTGCATTC) and, as template, the orangutan BAC clone CH276-344O16 whose BESs were mapped, by BLAT, on the opposite sides of the canonical centromere of PPY12 (ponAbe2 assembly).

### Gene expression analysis

RNA from PAB-9 and PPY-15 lymphoblastoid cell lines was extracted and retrotranscribed using RNeasy Mini kit and QuantiTect Reverse Transcription kit (Quiagen, Hilden, Germany), respectively. The expression profile of *SLC6A15* was evaluated by RT-qPCR experiments with SYBR Green (FastStart Essential DNA Green Master, Roche, Basel, Switzerland) on LightCycler^®^ 96 System (Roche). PAB-9 was used as the calibrator and *GAPDH* as reference. The PCR conditions were as follows: 10 min at 95 °C; followed by 45 cycles of 10 s at 95 °C, 10 s at 60 °C, and 10 s at 72 °C. Finally, to produce the melt curve, the PCR products were exposed to a temperature gradient from 65 °C to 95 °C. All measurements were performed in triplicate. Statistical significance was analyzed by using the LightCycler^®^ 96 Software 1.1 (Roche).

### Compaction analysis and statistical inference

Interprobe distances (d^2^) between BACs CH276-136P13 and CH276-12M5 were measured in pixels and converted in micrometers by considering: the micron pixel size of the CCD camera, the capturing binning (2 × 2), and the microscope objective. Each distance was then normalized to its nuclear radius (r^2^), measured from the DAPI stained area and transformed to the radius of a circle of the same area (Area = πr^2^). Statistical significance of differences between sets (n = 100) of normalized distances was assessed using the two-sided Mann-Whitney U test.

## Additional Information

**Accession codes:** Arrays data of CHIP-on-chip have been deposited to the NCBI’s Gene Expression Omnibus and are accessible through GEO Series accession number GSE81003. (https://www.ncbi.nlm.nih.gov/geo/query/acc.cgi? acc=GSE81003). PacBio805 has been submitted to GenBank under the Accession Number KX224531. NGS reads of the orangutan PPY-15 are available at http://www.ebi.ac.uk/ena/data/view/PRJEB13951. The NGS12 assembly is available at http://www.ebi.ac.uk/ena/data/view/LT571452-LT571452. The 106 paired-ends of the LR-PCR products have been submitted to GenBank under the Accession Number KX243426 - KX243531.

**How to cite this article**: Tolomeo, D. *et al*. Epigenetic origin of evolutionary novel centromeres. *Sci. Rep.* **7**, 41980; doi: 10.1038/srep41980 (2017).

**Publisher's note:** Springer Nature remains neutral with regard to jurisdictional claims in published maps and institutional affiliations.

## Supplementary Material

Supplementary Information

Supplementary Table S2
