# Supplementary material for: Epigenetic origin of evolutionary novel centromeres
Source: Sci Rep. 2017 Feb 3;7:41980. doi: 10.1038/srep41980 (PMC5290474; doi:10.1038/srep41980)
Supplement: Supplementary Information [file srep41980-s1.pdf]

Epigenetic origin of evolutionary novel centromeres

Doron Tolomeo[1,†], Oronzo Capozzi[1, †], Roscoe R. Stanyon[2], Nicoletta Archidiacono[1], Pietro D'Addabbo[1], Claudia R. Catacchio[1], Stefania Purgato[3], Giovanni Perini[3], Werner Schempp[4], John Huddleston[5-6], Maika Malig[5], Evan E. Eichler[5-6] and Mariano Rocchi[1,*]

[1]Department of Biology, University of Bari, Bari, Italy, [2]Department of Biology, University of Florence, 50122 Florence, Italy, [3]Department of Pharmacy and Biotechnology, University of Bologna, Bologna, Italy, [4]Institute of Human Genetics, University of Freiburg, 79106 Freiburg, Germany, [5]Department of Genome Sciences, University of Washington School of Medicine, Seattle, WA 98195, USA, [6]Howard Hughes Medical Institute, University of Washington, Seattle, WA 98195, USA

* Correspondence to mariano.rocchi@uniba.it

† these authors contributed equally to this work

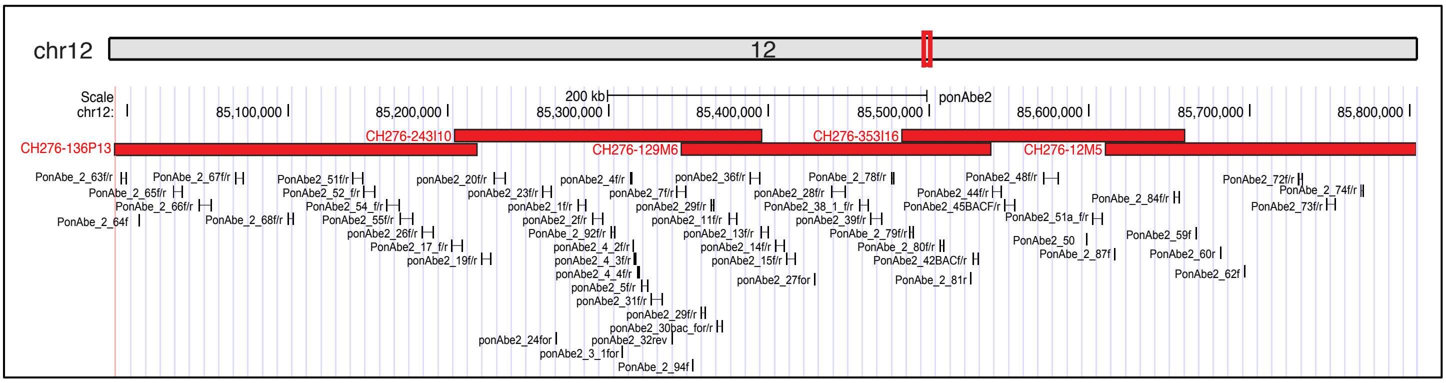

Supplementary Figure S1. Distribution of Sanger sequenced LR-PCR ends (black vertical bars) with respect to the ponAbe2 sequence of chromosome 12 and to the 5 sequenced BACs of the CH276 library (red rectangles). Some ends are not reported because of their low quality. On the left of each LR-PCR product are reported the corresponding primer names.

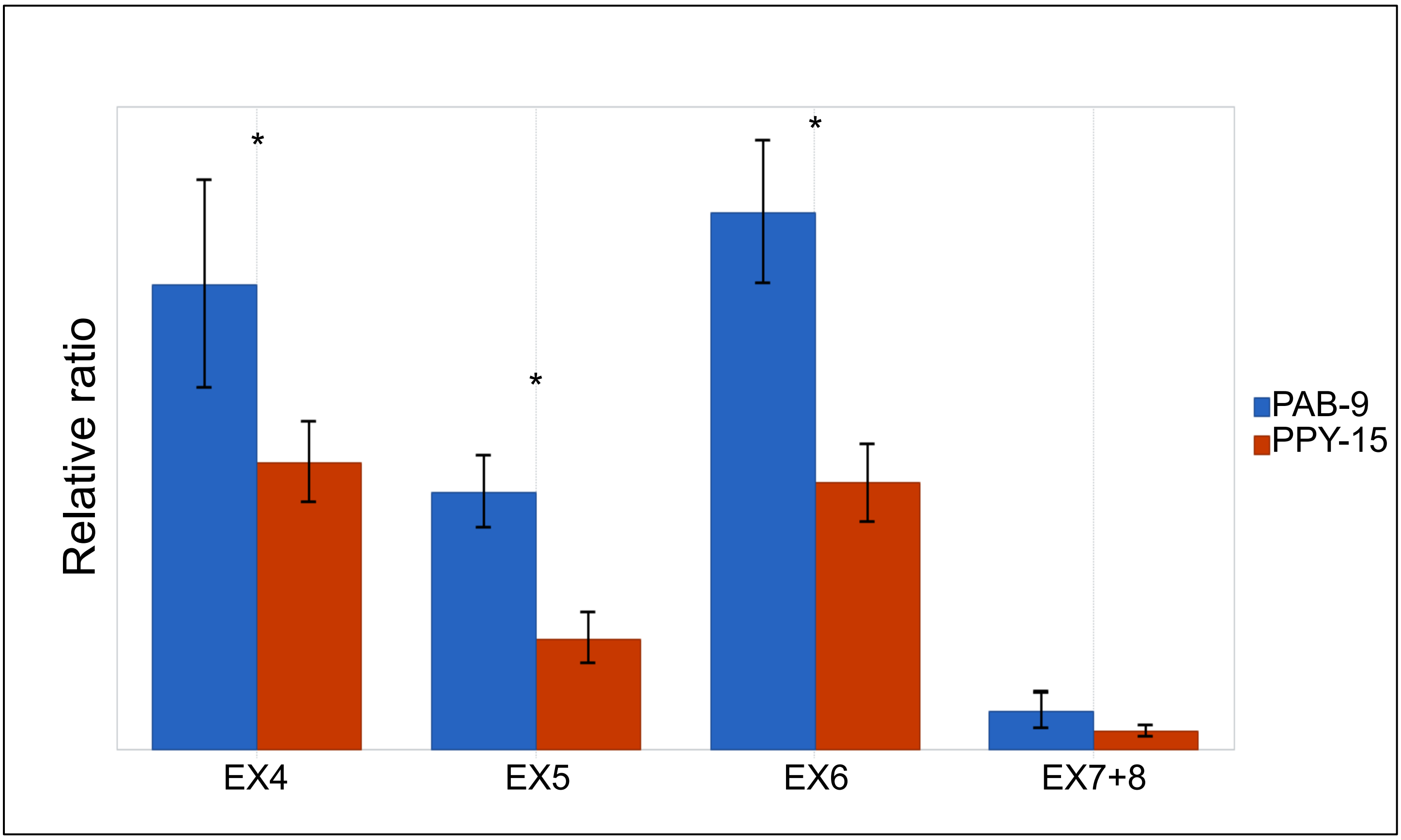

Supplementary Figure S2. Exon-specific reverse transcription quantitative PCR analysis of *SLC6A15*, performed in PPY-15 vs PAB-9. The statistical analysis was carried out using the LightCycler® 96 Software 1.1 (Roche). Asterisks indicate statistically significant results ($p < 0.05$).

primer forward name	sequence (5'-3')	map position (ponAbe2)	alignment to PacBio810 sequence	primer reverse name	sequence (5'-3')	map position (ponAbe2)	alignment to PacBio810 sequence	Long-range PCR results	5' end Sanger sequencing	% identity vs PacBio810 sequence	% identity vs ENC Illumina sequence	Sanger sequ	% identity vs PAC BIO sequence	% identity vs neo12seq
PonAbe_2_63_for	GGTGATCCTGACAACTCCCA	chr12:84995606-84995625	✔	*PonAbe_2_63_rev*	CACTGGGCTATGACATTGGC	chr12:84999186-84999205	✔	✔	✔	99 399	99 900	✔	98 951	99 894
PonAbe_2_64_for	AGCTGAACTGATGGAGGCTT	chr12:85007334-85007353	✔	*PonAbe_2_64_rev*	TGTCTCCAGGTCCCACATTC	chr12:85011380-85011399	✔	✔	✔	98 605	99 034	✗		
PonAbe_2_65_for	ATGCACACCCTTGGGTCATA	chr12:85028799-85028818	✔	*PonAbe_2_65_rev*	ACCATCATGCCACAGGAGAA	chr12:85034227-85034246	✔	✔	✔	99 779	100 000	✔	99 433	100 000
PonAbe_2_66_for	GATTTGAACAGCTGTGCCCA	chr12:85044617-85044636	✔	*PonAbe_2_66_rev*	GAGTACTTCCCACTGGCCTT	chr12:85052834-85052853	✔	✔	✔	98 529	99 628	✔	98 566	99 618
PonAbe_2_67_for	TGAGATGGTGGCCTCTTTGT	chr12:85067043-85067062	✔	*PonAbe_2_67_rev*	CTACTTGGGGAGGCCTTTGA	chr12:85072699-85072718	✔	✔	✔	99 793	100 000	✔	99 165	99 687
PonAbe_2_68_for	CACGCAAAAGTTCCAGAGCT	chr12:85099907-85099926	✔	*PonAbe_2_68_rev*	ATTACACTCCTGGCCCTTCC	chr12:85103355-85103374	✔	✔	✔	99 119	99 804	✔	99 176	99 691
PonAbe2_51_for	CTGCTCAAAAACGTCCATCA	chr12:85140268-85140287	✔	PonAbe2_51_rev	TCCATCTGGTCTTTCCCTTG	chr12:85147410-85147429	✔	✔	✔	99 685	99 790	✔	99 560	99 560
PonAbe2_52_for	CAAGGGAAAGACCAGATGGA	chr12:85147410-85147429	✔	PonAbe2_52_rev	ACTGCACCTCCCATACTTGG	chr12:85154536-85154555	✔	✔	✔	99 212	99 550	✔	99 839	99 839
PonAbe2_53_for	TCATCTGGGACAAACACCAA	chr12:85154638-85154657	✔	*PonAbe2_53_rev*	ATGCGTGAACTGCGTCATAG	chr12:85161861-85161880	✔	✔	✗			✗		
PonAbe2_54_for	CGCAGTTCACGCATCTCTTA	chr12:85161867-85161886	✔	PonAbe2_54_rev	GGCAGAACAAGGAGCAACTC	chr12:85169835-85169854	✔	✔	✔	99 570	99 677	✔	99 217	99 217
PonAbe2_55_for	GCTCCTTGTTCTGCCACTTT	chr12:85169840-85169859	✔	PonAbe2_55_rev	ACTATTGACCAGGGCCAGTG	chr12:85178134-85178153	✔	✔	✗			✗		
PonAbe2_55_for	AAGGGGAGCATCACACATCA	chr12:85169840-85169859	✔	PonAbe2_55_rev	ACAACACTCACCAAACCAGC	chr12:85178134-85178153	✔	✔	✔	99 320	99 434	✔	99 201	99 799
PonAbe2_56_for	CACTGGCCCTGGTCAATAGT	chr12:85178134-85178153	✔	PonAbe2_56_rev	ATGAACTAGGCAGGCAAGGA	chr12:85183159-85183178	✔	✔	✗			✗		
ponAbe2_26_for	CACACTGAGCTGCGAATCAT	chr12:85183658-85183678	✔	ponAbe2_26_rev	GGGGGATTTTGGTTTTCTGT	chr12:85190875-85190894	✔	✔	✔	99 796	99 796	✔	99 406	99 703
ponAbe2_25_for	TCAGGGCCAAGAGACAGAAT	chr12:85191066-85191086	✔	ponAbe2_25_rev	GCTAAGGTTCAGAGCCACCA	chr12:85200747-85200767	✔	✔	✗			✗		
PonAbe2_17_for	AGGGAACAACAGCAAAATGG	chr12:85202147-85202167	✔	ponAbe2_17_rev	TCCAGGTAATCCACCCTCAC	chr12:85209202-85209222	✔	✔	✔	98 758	98 755	✔	98 291	98 575
PonAbe2_18_for	TGGAGGAGGGATGAGTGTTC	chr12:85211551-85211571	✔	ponAbe2_18_rev	TGTGAACCCCGAAGATGAAT	chr12:85218575-85218595	✔	✔	✗			✗		
ponAbe2_19_for	GTCACTGCCCAATGTTTCCT	chr12:85221148-85221168	✔	ponAbe2_19_rev	TTTCGTTCACATCAGGGTCA	chr12:85227147-85227167	✔	✔	✔	99 270	99 582	✔	99 880	100 000
ponAbe2_20_for	TGGGAAAATGGAATGTCCTC	chr12:85228426-85228445	✔	ponAbe2_20_rev	TGCAACTTCCTCAAGCAAAA	chr12:85236378-85236397	✔	✔	✔	98 337	98 337		99 248	99 678
ponAbe2_21_for	GCTGGACTGACTCTGGAAGG	chr12:85238636-85238656	✔	ponAbe2_21_rev	TAGCTCATGGGACGCTAACC	chr12:85246351-85246371	✔	✔	✗			✗		
ponAbe2_22_for	CGGGAATGAACAAAGAAAGG	chr12:85251212-85251232	✔	ponAbe2_22_rev	TTGCATCTGTGGACTTGCAT	chr12:85257801-85257821	✔	✔	✗			✗		
ponAbe2_23_for	ACAGCTTTCCCCACAACATC	chr12:85258634-85258654	✔	ponAbe2_23_rev	TGGCTTTCATTTTCCACTCC	chr12:85264672-85264692	✔	✔	✔	99 635	100 000	✔	99 394	100 000
ponAbe2_24_for	GACAGGACTGGATGCGATTT	chr12:85267278-85267298	✔	ponAbe2_24_rev	AAGAAAAGGCCCAACACTGA	chr12:85274519-85274539	✔	✔	✔	99 815	100 000	✗		
ponAbe2_1_for	TTCCACTCGAAGCTTTAGGAAG	chr12:85281319-85281341	✔	ponAbe2_1_rev	GTGCCATTCCATACTTCTGACA	chr12:85286537-85286559	✔	✔	✔	99 897	100 000	✔	99 695	99 695
ponAbe2_2_for	ATCCCAGCTGATAGCAGAGAAG	chr12:85290224-85290246	✔	ponAbe2_2_rev	TATGTCAGGCACTGTGAAATCC	chr12:85297055-85297077	✔	✔	✔	98 312	99 158	✔	100 000	100 000
ponAbe2_33_for	CCACTGCTAGTTGGCTCTCC	chr12:85296690-85296710	✔	ponAbe2_33_rev	GGGAAAGGATGGACAGATGA	chr12:85304691-85304711	✔	✔	✗			✗		
PonAbe_2_92_for	AGCAATAACGGGTGTCCAGA	chr12:85301787-85301806	✔	*PonAbe_2_92_rev*	GGGTGGAGGACATGACTGAA	chr12:85304310-85304329	✔	✔	✔	99 254	99 574	✔	99 228	99 449
ponAbe2_3_for	TGAAGCAAAGGCAGGGTATATT	chr12:85302239-85302261	mismatches	ponAbe2_3_rev	TTAGGATCAAGACGAGGGTGAT	chr12:85309005-85309027	✔	✗						
ponAbe2_35_for	CCCACATTTGCAGTTGAATG	chr12:85380587-85380606	✔	ponAbe2_35_rev	CTTTGGTGACCTGGTCCTGT	chr12:85390101-85390121	no matches	✗						
ponAbe2_34_for	TCTGTCCATCCTTTCCCACT	chr12:85304694-85304714	mismatches	ponAbe2_3_rev	TTAGGATCAAGACGAGGGTGAT	chr12:85309005-85309027	✔	✗						
ponAbe2_3_1_for	CCAGACATGCAAACAACAGG	chr12:85308631-85308650	✔	ponAbe2_4_1_rev	GCCACTTTGGAAATACGATCA	chr12:85314977-85314998	✔	✔	✔	99 214	99 476	✗		
ponAbe2_4_for	AGATCCCTTCAATTTGTGCAGT	chr12:85313576-85313598	✔	ponAbe2_4_1_rev	GCCACTTTGGAAATACGATCA	chr12:85314977-85314998	✔	✔	✔	99 001	99 800	✔	98 800	99 800
ponAbe2_4_2_for	ATGAGAGGAACCCAGAAATGTG	chr12:85315017-85315038	✔	ponAbe2_4_2_rev	CTTCCAAGGTGTTGAGCCATA	chr12:85316196-85316216	✔	✔	✔	98 812	99 901	✔	98 915	99 803
ponAbe2_4_3_for	GGCTAAAGAGGGTTCTCAAATG	chr12:85316307-85316328	✔	ponAbe2_4_3_rev	CCCACTCAAGGGCTGAAATA	chr12:85317579-85317598	✔	✔	✔	98 234	99 902	✔	98 127	99 719
ponAbe2_4_4_for	GACTGAAGCAAAACCTGGGTA	chr12:85318080-85318100	✔	ponAbe2_4_rev	TACTTCCCTTGAATTTGCTGGT	chr12:85319607-85319629	✔	✔	✔	98 793	99 596	✔	99 417	99 708
ponAbe2_5_for	TGAGCTTAGACCCAAGAAGGAG	chr12:85320416-85320438	✔	ponAbe2_5_rev	AGGTAAGGCAAAGATGGAAACA	chr12:85325396-85325418	✔	✔	✔	97 730	99 242	✔	99 610	99.74
ponAbe2_31_for	AGGCTGAAACAGCTGGAAAA	chr12:85326360-85326380	✔	ponAbe2_31_rev	TGAACAGCTTTGATCGTTGC	chr12:85334103-85334123	✔	✔	✔	99 048	99 259	✔	99 164	99 164
ponAbe2_6_for	AGTGAAAAACCTTGAGGGTTCA	chr12:85331853-85331875	✔	ponAbe2_6_rev	TGCATTGACTTGAAGTTTTGCT	chr12:85338164-85338186	mismatches	✗						
ponAbe2_32_for	GACTGCATCTGACTGGCAAA	chr12:85333669-85333689	✔	ponAbe2_32_rev	GGGGGAACTTCCACGATAAT	chr12:85340093-85340113	✔	✔	✗			✔	100 000	100 000
ponAbe2_7_for	TCACTCCCTTTGCCATAGTTTT	chr12:85342564-85342586	✔	ponAbe2_7_rev	GTGCATGTAGAAGGATTGGTGA	chr12:85348961-85348983	✔	✔	✔	100 000	100 000	✔	99 077	99 471
PonAbe_2_94_for	AATGGCTTGGACATGGGAGA	chr12:85352567-85352586		*PonAbe_2_94_rev*	TGATCTTGAACCACCCAGCT	chr12:85356056-85356075	✔	✔	✔	99 000	99 667	✗		
ponAbe2_8_for	TACCTGGGACTACAGGAAAGGA	chr12:85353748-85353770	✔	ponAbe2_8_rev	TACCTGGGACTACAGGAAAGGA	chr12:85359951-85359973	✔	✔	✗			✗		
ponAbe2_29_for	AGCATTTGGAGGCAAAAAGA	chr12:85357504-85357524	✔	ponAbe2_29_rev	AAGGTAACAAGCCAGGCAAA	chr12:85364351-85364371	✔	✔	✔	99 579	99 579	✔	99 697	99 798
PonAbe_2_30bac_for	GCACTACCACTGGCAAATGT	chr12:85360178-85360197	✔	ponAbe2_30_rev	CTGCGGAAACAGCCATATTT	chr12:85368488-85368508	✔	✔	✔	98 492	99 296	✔	99 591	99 796
ponAbe2_10_for	CCGGACATCACAAGGAGACT	chr12:85362387-85362407	✔	ponAbe2_10_rev	CAGAAAATACAAAGTGGGGATG	chr12:85374094-85374116	✔	✔	✗			✗		
ponAbe2_30_for	GGCATGGCAGGAAAATTAAA	chr12:85364281-85364301	incorrect orientation	ponAbe2_30_rev	CTGCGGAAACAGCCATATTT	chr12:85368488-85368508	incorrect position	✗						
PonAbe2_10_1_for	TAACAACTGGTGGCCCGTAT	chr12:85365191-85365210	incorrect orientation	PonAbe2_10_1_rev	GCTGTGAGAGTGATGGCATG	chr12:85371406-85371425	incorrect orientation	✗						
PonAbe_2_77_for	TAACAACTGGTGGCCCGTAT	chr12:85365191-85365210	✔	*PonAbe_2_77_rev*	GCTGTGAGAGTGATGGCATG	chr12:85371406-85371425	✔	✔	✔	99 769	99 769		99 896	100 000
ponAbe2_9_for	TTCCTCAACACAAGCGAATG	chr12:85368347-85368367	incorrect orientation	ponAbe2_9_rev	AATTCCTCAATGGCTCTCCA	chr12:85367730-85367750	incorrect orientation	✗						
ponAbe2_11_for	CACAATTCCATGCAGCTTTC	chr12:85375091-85375111	✔	ponAbe2_11_rev	CCAGGAGAGGTTGCACATTT	chr12:85380651-85380671	✔	✔	✔	99 591	99 591	✔	99 643	99 465
PonAbe2_12_1_for	GCCTCTTTCGTGGTCTTTCC	chr12:85380672-85380691	✔	*PonAbe2_12_1_rev*	CCTAGAACAGCCAGTGACCA	chr12:85387017-85387036	✔	✔	✗			✗		
ponAbe2_36_for	AAAGCAAGTGGCATCTTCGT	chr12:85387977-85387997	✔	ponAbe2_36_rev	GTCGTTTGGGAAAACTTCCA	chr12:85395319-85395339	✔	✔	✔	99 605	100 000	✔	99 775	99 775
ponAbe2_13_for	GTTTTCCCAAACGACATTGG	chr12:85395324-85395344	✔	ponAbe2_13_rev	GCTGTGCATCTCCTGTTGAA	chr12:85382374-85400386	✔	✔	✗	99 404	99 404	✗	99 579	99 789
ponAbe2_14_for	TTTGCCTCATGGAAGTCTCC	chr12:85404115-85404135	✔	ponAbe2_14_rev	AGAAGAAAGGACCGTGGTTG	chr12:85410284-85410304	✔	✔	✗	99 893	99 893	✗	99 901	99 901
ponAbe2_15_for	CTGAAGTGCTGTACCGTGGA	chr12:85410838-85410858	✔	ponAbe2_15_rev	TAGGGGACCATCCAAGTCTG	chr12:85416917-85416937	✔	✔	✔	100 000	100 000	✔	98 824	98 824
ponAbe2_16_for	GTTGGAGCAAAGTGCGAAAC	chr12:85422670-85422690	✔	ponAbe2_16_rev	ACAGAAGCAAGGGTGAGGAG	chr12:85428522-85428542	✔	✔	✗			✗		
ponAbe2_27_for	CTCCTCACCCTTGCTTCTGT	chr12:85428522-85428542	✔	ponAbe2_27_rev	ATGGACTGTGAGCTCCTGCT	chr12:85436714-85436733	✔	✔	✔	99 288	100 000	✗		
ponAbe2_28_for	TTTTGGTGGTGACAGTGCAT	chr12:85439387-85439407	✔	ponAbe2_28_rev	GACTGTGACGGGAGGGATAA	chr12:85448275-85448295	✔	✔	✔	99 623	99 623	✔	99 768	99 768
ponAbe2_37_for	AAGGCAGGTAGCAGGTCTCA	chr12:85448477-85448497	✔	ponAbe2_37_rev	TCAAACCTTTTGCAGGCTCT	chr12:85456683-85456703	✔	✔	✗			✗		
ponAbe2_38_for	AGAGCCTGCAAAAGGTTTGA	chr12:85456683-85456703	✔	ponAbe2_38_rev	TACTCACCTCCCACCTCACC	chr12:85466844-85466863	no matches	✗						
PonAbe2_38_1_for	TTTCCAGAGCGTTGCACAAA	chr12:85456925-85456944	✔	*PonAbe2_38_1_rev*	GTTTGCAGTTCTGGGCTTCA	chr12:85462890-85462909	✔	✔	✔	99 021	99 021	✔	97 708	97 708
ponAbe2_39_for	TTCAGATGAGCCATGTGTGG	chr12:85463799-85463819	✔	ponAbe2_39_rev	AGTGCCCCTTATTGTGTTGC	chr12:85471109-85471129	✔	✔	✔	99 189	99 493	✔	99 489	99 898
PonAbe_2_78_for	TACTTTCCTCACGCGTCCTT	chr12:85476239-85476258	✔	*PonAbe_2_78_rev*	TCCTGGTGTTGGTGTATGCT	chr12:85478909-85478928	✔	✔	✔	99 209	99 505		99 453	99 781
PonAbe_2_79_for *	GGTCTGTCTGGGTGGTTGTA	chr12:85488136-85488155<br>chr12:85492079-85492098	✔	*PonAbe_2_79_rev* *	TTTCCCTCCATTCCCCACTC	chr12:85490830-85490849<br>chr12:85494685-85494704	✔	✔	✔	98 565	99 522	✔	99 698	100 000
PonAbe_2_80_for	TCTGCCCTGTGTCAAGTGAT	chr12:85507199-85507218	✔	*PonAbe_2_80_rev*	CAGAGACGCGAGGTGAATTG	chr12:85509959-85509978	✔	✔	✔	99 538	100 000		99 196	99 799
PonAbe2_42_for	TCTTTGTCTTCCCCTTCCCC	chr12:85523600-85523619	✔	*PonAbe2_42BAC_rev*	ACCACCGATCCCACAGAAAT	no matches on ENC region	✔	✔	✗			✗		
PonAbe2_42_for	TCTTTGTCTTCCCCTTCCCC	chr12:85523600-85523619	too distantly positioned	PonAbe2_42_rev	TTCTGTCTGGGAGATCTGGC	chr12:85531041-85531060	too distantly positioned	✗	✗					
PonAbe_2_81_for	CCCAGGGAAGACTTTGGCTA	chr12:85523703-85523722	✔	*PonAbe_2_81_rev*	CCAAAGACTACCAGGCCAGA	chr12:85526927-85526946	✔	✔	✗			✔	99 284	99 898
PonAbe2_42BAC_for	CAAAGTGCTCTGGGGTCCTA	no matches on + strand	✔	PonAbe2_42_rev	TTCTGTCTGGGAGATCTGGC	chr12:85531041-85531060	✔	✔	✔	99 024	99 024		99 257	99 894
PonAbe2_43_for	GCCAGATCTCCCAGACAGAA	chr12:85531041-85531060	✔	PonAbe2_43_rev	CCACTCCCATCCCAGACAAA	chr12:85539207-85539226	✔	✔	✗					
PonAbe2_44_for	GGAGTGGGGAGGACAAGTTT	chr12:85539220-85539239	✔	PonAbe2_44_rev	AGTGCTTTACAGTTGCGTCT	chr12:85545319-85545338	✔	✔	✔	99 322	99 322	✔	99 225	99 483
PonAbe2_45_for	TGTTTAGACGCAACTGTAAAGCA	chr12:85545314-85545336	too distantly positioned	PonAbe2_45_rev	AGGATTTCTGAAGCTGGCCT	chr12:85554026-85554045	too distantly positioned	✗	✗					
PonAbe2_45BAC_for	ACCGCTTTACTTCTGCTCCT	no matches	✔	PonAbe2_45_rev	AGGATTTCTGAAGCTGGCCT	chr12:85554026-85554045	✔	✔	✔	97 838	97 838	✔	98 723	98 936
PonAbe2_48_for	GGCCTTGTCCCCTACTCTTT	chr12:85571582-85571601	✔	PonAbe2_48_rev	ATGACCATGCCTCAGGAACA	chr12:85581342-85581361	✔	✔	✔	100 000	99 848	✔	99 593	99 593
PonAbe2_49_for	ACCTGCAATTTCTGTCTCCC	chr12:85581254-85581273	✔	PonAbe2_49_rev	GCAGTTCCTTCCCTTACCCT	chr12:85586250-85586269	✔	✔	✗					
PonAbe2_50_for	CTCCCGAAGTGCTTACATGC	chr12:85592003-85592022	✔	PonAbe2_50_rev	TGTCCATCGTGAGCTGACTT	chr12:85598954-85598973	✔	✔	✗			✔	98 936	99 409
PonAbe2_51a_for	TTAACGACAGGGACAGGACC	chr12:85601856-85601875	✔	PonAbe_2_51a_rev	GGGCAGAGGAGTGAGAGTTT	chr12:85609057-85609076	✔	✔	✔	99 482	99 485	✔	99 341	99 890
PonAbe_2_87_for	ATTGGCTCATGTCCAGGTCA	chr12:85617025-85617044	✔	*PonAbe_2_87_rev*	AGCTTGACCCAGCCTAACAT	chr12:85621477-85621496	✔	✔	✔	99 825	100 000	✗		
PonAbe_2_84_for	CCACAGGCTAGGGAAGTTCA	chr12:85653084-85653103	✔	*PonAbe_2_8_rev*	ACTCTCCAGGACGTAGGACT	chr12:85656562-85656581	✔	✔	✔	99 402	99 601	✓	99 874	100 000
PonAbe2_59_for	AGGGAGACAGAATGTGGTGG	chr12:85666829-85666848	✔	*PonAbe2_59_rev*	AATCCTGCAACACACACACC	chr12_random:9590779-9590798	✔	✔	✔	99 153	99 576	✔	low quality	
PonAbe2_60_for	GGTCATCAGCTGCATCCATG	chr12:85677242-85677261	✔	PonAbe2_60_rev	GGTTCCTGGTTCTACTGGCT	chr12:85682728-85682747	✔	✔	✗			✗	98 930	99 287
PonAbe2_61_for	CAGAGCACGCATGTCTTCTC	chr12:85685010-85685029	✔	*PonAbe2_61_rev*	CAAGTCCCGAGTTTGCACAA	chr12:85692242-85692261	✔	✔	✗			✗		
PonAbe2_62_for	CGTGTGGCATGTGTGAGAAA	chr12:85696941-85696960	✔	*PonAbe2_62_rev*	AAGTGGGGTGGGAAGAGATG	chr12:85703900-85703919	✔	✔	✔	99 027	99 459		low quality	
PonAbe _2_72_for	ATCCAAACTCCCGCCTGTTA	chr12:85731875-85731894	✔	*PonAbe _2_72_rev*	TGAGTGGAGAGACAGCACTG	chr12:85735256-85735275	✔	✔	✔	97 823	99 230	✔	98 957	100 000
PonAbe2_73_for	GAAGGAGGCTATAGGGGCAG	chr12:85748124-85748143	✔	*PonAbe2_73_rev*	GGGGATGAGGAGGAAGTCAG	chr12:85754284-85754303	✔	✔	✔	99 389	99 695	✔	99 691	100 000
PonAbe_2_74_for	TGTGACTAGTGCCGTGATGT	chr12:85768569-85768588	✔	*PonAbe_2_74_rev*	CCTCTGACCAAGGCACAAAC	chr12:85771584-85771603	✔	✔	✔	97 593	99 799	✔	99 676	99 784

Supplementary Table S1. Primers used for LR-PCR. Primers designed on PacBio805 sequence are reported in italic. * = sequence duplicated on PonAbe2 assembly; ✓ = positive result; ✗= negative result.

primer name	sequence (5'-3')	map position (ponAbe2)
SLC15_4F	TGGAACTCTCTGTGGGTCAA	chr12:85,479,014-85,479,033
SLC15_4R	CCATACACCAATGCTGCCTC	chr12:85,478,984-85,479,003
SLC15_5F	CTCAGTCTTTTCAGCAACCCC	chr12:85,478,505-85,478,525
SLC15_5R	TTTCACCAAAGGACACTGATCC	chr12:85,478,476-85,478,497
SLC15_6F	GGGCTTAAACTGGAAGATGACC	chr12:85,476,967-85,476,988
SLC15_6R	CAAGCAAACCATGACCCAGG	chr12:85,476,931-85,476,950
SLC15_7F	TCGCCACATGTTTACCCCTA	chr12:85,465,882-85,465,901
SLC15_8R	GCAGCTTCTCTCCAGACCTT	chr12:85,463,057-85,463,076

Supplementary Table S3. Primers used for RT-qPCR of the *SLC6A15* gene.
